# Microbes of traditional fermentation processes as synthetic biology chassis to tackle future food challenges

**DOI:** 10.3389/fbioe.2022.982975

**Published:** 2022-09-16

**Authors:** Adán Andrés Ramírez Rojas, Razan Swidah, Daniel Schindler

**Affiliations:** Max Planck Institute for Terrestrial Microbiology, Marburg, Germany

**Keywords:** synthetic biology, microbe domestication, microbial chassis, fermenation, future food, biological materials, yeast

## Abstract

Microbial diversity is magnificent and essential to almost all life on Earth. Microbes are an essential part of every human, allowing us to utilize otherwise inaccessible resources. It is no surprise that humans started, initially unconsciously, domesticating microbes for food production: one may call this microbial domestication 1.0. Sourdough bread is just one of the miracles performed by microbial fermentation, allowing extraction of more nutrients from flour and at the same time creating a fluffy and delicious loaf. There are a broad range of products the production of which requires fermentation such as chocolate, cheese, coffee and vinegar. Eventually, with the rise of microscopy, humans became aware of microbial life. Today our knowledge and technological advances allow us to genetically engineer microbes - one may call this microbial domestication 2.0. Synthetic biology and microbial chassis adaptation allow us to tackle current and future food challenges. One of the most apparent challenges is the limited space on Earth available for agriculture and its major tolls on the environment through use of pesticides and the replacement of ecosystems with monocultures. Further challenges include transport and packaging, exacerbated by the *24/7 on-demand* mentality of many customers. Synthetic biology already tackles multiple food challenges and will be able to tackle many future food challenges. In this perspective article, we highlight recent microbial synthetic biology research to address future food challenges. We further give a perspective on how synthetic biology tools may teach old microbes new tricks, and what standardized microbial domestication could look like.

## Introduction

Synthetic biology is a discipline of biology which aims to domesticate and standardize DNA parts, modularize cellular processes on regulatory and functional level, and ultimately, aims to construct synthetic organisms from scratch serving as chassis in application based processes (Wang et al., 2018; Ostrov et al., 2019; Schindler, 2020). In recent years, many technological advances have been made, especially towards the design, synthesis and construction of synthetic DNAs up to whole genome size (Schindler et al., 2018; Ostrov et al., 2019). However, building designer organisms from scratch is still limited due to the compulsory large-scale DNA synthesis and extensive genetic engineering during the “debugging” phase, as well as the lack of understanding of what the essential components supporting a synthetic minimal life are. Minimal chassis would be outstanding tools for researchers, facilitating testing of parts and pathways within a minimal cellular metabolism while causing minimum interference. However, it will take more time until such tools for systematic analysis are available. On the other hand, cell-free biology using either cell extracts or the essential purified proteins and cofactors has become popular (Shimizu et al., 2005; Swartz, 2006). Researchers are able to produce valuable compounds in cell-free systems but the method currently lacks scalability (Dondapati et al., 2020).

Today, a broad spectrum of products are generated by microbes including engineered microbes in industrialized processes. Here, often random mutagenesis and Adaptive Laboratory Evolution (ALE) are tools employed to optimize strains, but evolution is not always beneficial, strains are constantly evolving to minimize cellular burden which may cause scale-up process to fail (Schmidt, 2005; Ellis, 2019). Bearing this in mind, the diversity of currently industrially used microbial chassis is rather low in comparison to the actual microbial biodiversity. In many cases there are unexplored wild-type microorganisms which may perform as well or even better for a given product compared to engineered model organisms. Researchers need to move away from the dichromatic view of model and non-model organisms to appreciate the whole diversity available for application. Now that most laboratories can perform whole genome sequencing and produce high quality reference genomes of microbes using combinations of short- and long-read sequencing techniques, we are not limited to the use of a handful of model organisms, and are able to expand into use of non-model microorganisms. Further advances of synthetic biology tools such as standardized DNA assembly and innovative genome manipulation tools allow the quick establishment of new microbial systems. In addition to this, many high-throughput technologies are broadly available for in-depth characterization, such as transcriptome, proteome and metabolome profiling allowing researchers to accumulate large-scale datasets with the potential to create whole-cell models (Sun et al., 2021; Lu et al., 2022). This is due to the efforts of interdisciplinary work between experimentalists, data scientists and software engineers to constantly improve software tools, many of which are now able to be used in a plug-and-play manner by any user.

**FIGURE 1:**
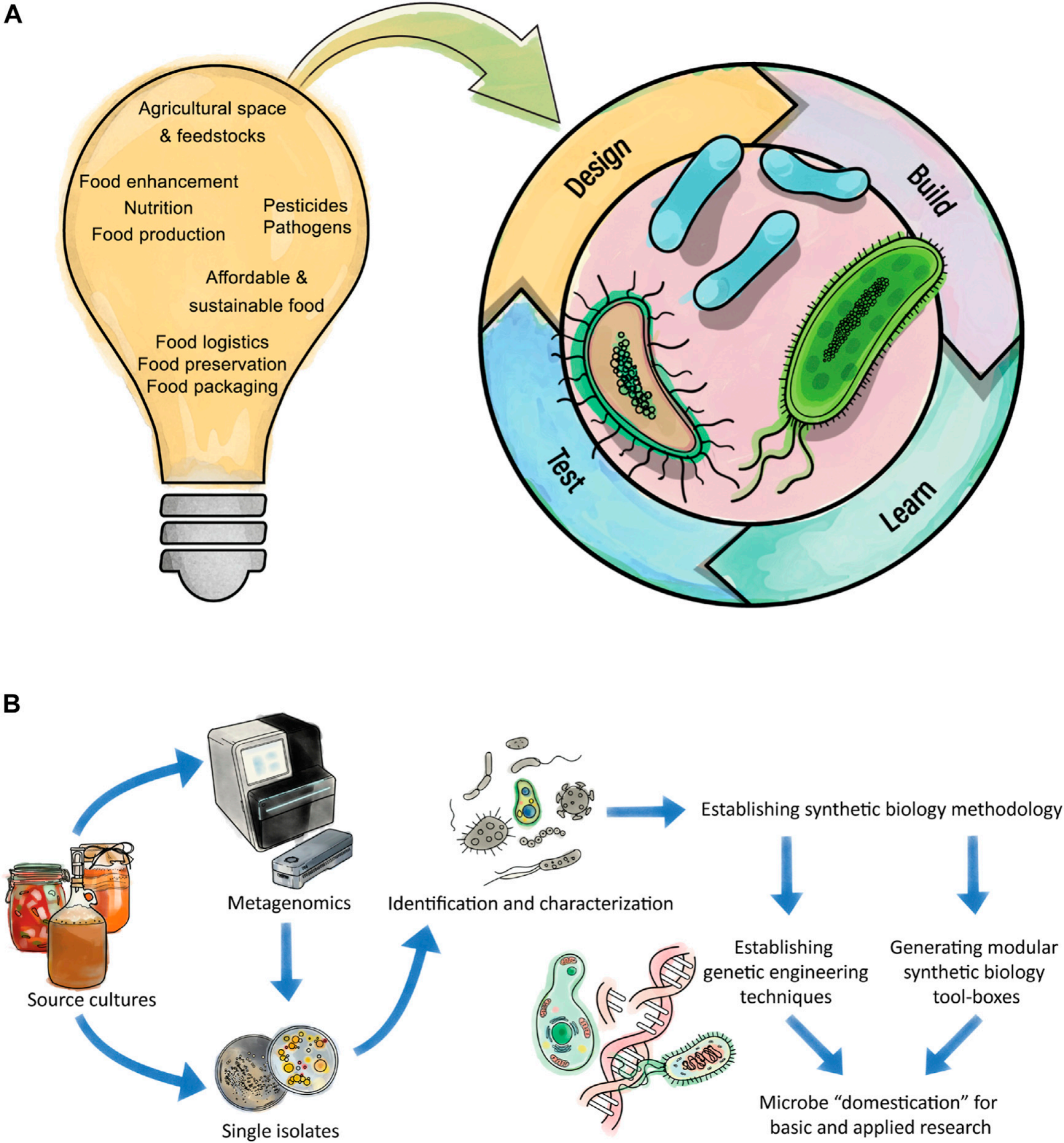
Challenges of future foods which give rise to opportunities for synthetic biology. **(A)** Synthetic biology tries to implement engineering principles into life. The lightbulb highlights some of the challenges for future foods. These challenges may be inspirational for experimental designs for synthetic biology methodology with the potential to improve a process or overcome related problems. Microbes can be altered through the “Design-Build-Test-Learn” cycle for a greater aim and particular microbes from traditional fermentation processes have the potential to address future food challenges. **(B)** Workflow showing a pipeline to domesticate microbes, for example from traditional fermentations processes. The initial source can be analyzed by traditional isolation of individual microbes or by metagenomics approaches to initially get an overview of the community before individuals are isolated. The isolated microbes need to be identified and characterized. Once the organism is known, one can start to make the organism accessible for synthetic biology approaches. Therefore, initial genetic engineering methods need to be established (i.e., transformation procedures), followed by advances in engineering tools (i.e., CRISPR/Cas-based methods) and the generation of modular tool-boxes for quick and reliable engineering of the organism. The established tools allow microbe domestication, for example by removal or addition of genes, for easier handling. Subsequently the domesticated microbe can be used for intensive engineering towards a desired goal for example the assimilation of a sustainable feedstock.

All of these advances allow researchers to tackle global challenges. One of the biggest challenges humankind is facing is how to guarantee a sustainable food supply for all humans on Earth (Searchinger et al., 2019). In recent times, this has become more apparent, indicated by the collapsing supply chains due to the SARS-CoV-2 pandemic and geopolitical instability (Barman et al., 2021; Moosavi et al., 2022). Presumably such factors will not decline and will instead become more evident in the light of climate change (IFPRI, 2022). Researchers, policy makers and politicians need to work together to identify solutions to how everyone on Earth can be supplied with sustainable food. However, these challenges extend beyond food production. For example, the preserving, packaging and logistics behind each product on the shelves are challenges but also opportunities which synthetic biology can use to make future foods more sustainable (Figure 1A).

Within the scope of this perspective we highlight how synthetic biology research can adapt microorganisms from traditional fermentation processes, making them accessible for synthetic biology workflows to obtain new models to tackle future food challenges. We highlight how microorganisms can help us to improve food quality, reduce environmental impact by local production and alternative feedstocks, and how microbes can be used to build biomaterials serving as sustainable packaging material. We further give opinions where we believe there are gaps and propose future directions researchers should investigate.

### Microbial organisms from traditional fermentation

Many daily products such as sourdough bread, coffee, and chocolate are produced by microorganisms in a process called fermentation (Marco et al., 2017; Dimidi et al., 2019). Since the work of van Loewenhook and Pasteur we have been aware that humans used and domesticated microbes for the preparation and preservation of food. The majority of organisms within fermentation processes are *generally recognized as safe* (“GRAS organisms”) and have the potential to be model systems. One disadvantage of fermented food over highly industrialized food is the rather time-consuming production process. However, it pays off with health benefits and complex flavors over highly industrialized products (Sanlier et al., 2019). Even though the procedure takes time it is scalable and one can find certain industrial products where fermentation was used to produce them for example raw apple cider vinegar, chocolate and coffee. With a rise of health awareness there is an increased interest from various companies towards traditional fermentation and products like sourdough bread and kombucha have become available to more consumers. Table 1 gives an overview of some fermented foods and the composition of the microbial community. Many of these cultures are domesticated from their original source for generations resulting in balanced communities with adaptation towards a respective condition or product. Often traditional fermentation cultures contain *Saccharomyces cerevisiae* strains and various lactic acid bacteria (LAB). Both are traditionally known for their roles in fermentation processes and are extensively explored in synthetic biology (Mays and Nair, 2018; De Filippis et al., 2020; Molinet and Cubillos, 2020; Schindler, 2020). However, within this perspective article, we would like to draw attention to some other, less explored microbes and their complex communities. Highly interesting microbes can be found in traditional farmhouse brewing. As an example, Kveik yeasts, which are a genetically distinct group of domesticated *S*. *cerevisiae* brewing yeasts, are highly temperature-tolerant, have an impressive fermentation speed and exceptionally high flocculation, resulting in crystal clear products within a short period of time (Krogerus et al., 2018; Preiss et al., 2018; Foster et al., 2022). At the same time, Kveik yeasts produce highly desirable flavor profiles during fermentation (Kawa-Rygielska et al., 2021; Luo et al., 2021; Kawa-Rygielska et al., 2022). Farmhouse brewing strains are often communities of various microbes (bacteria and yeasts) and not single strains which are used in industry. Microbial communities, especially from traditional fermentation processes, are treasure troves for potential new synthetic biology chassis based on their phenotypic properties. Systematic analysis of microbial communities from fermentation cultures is important to understand their composition (i.e. by metagenomics), their products (i.e. by metabolomics), and their ecology (Lavefve et al., 2019). With Next Generation Sequencing techniques being available to the majority of researchers, systematic studies of fermentative cultures are becoming more frequent (Pswarayi and Ganzle, 2019; Weckx et al., 2019; Arikan et al., 2020; Comasio et al., 2020; Fernandez-Nino et al., 2021).

**TABLE 1:** Overview of some traditional fermentation procedures and their features.

Product	Source*	Relevant identified microbes	Features	References
**Beverages**				
Beer	Barley or other cereals	*Saccharomyces cerevisiae, S. pastorianus, Lactobacillus* sp.*, Pediococcus* sp.	Top or bottom fermenting. Production of glycerol, vicinal diketones, alcohols, esters, organic acids.	Bokulich and Bamforth. (2013)
Wine	Grapes	*S. cerevisiae, Oenococcus oeni, Lactobacillus plantarum*	Alcoholic and malolactic fermentation. Production of aromatic compounds.	Comitini et al., (2021)
Kombucha	Tea leaves	*Komagataeibacter* sp.*, Gluconobacter oxydans, Zygosaccharomyces sp.*	Production of cellulose, antibacterial and antioxidant compounds.	Kluz et al. (2022)
**Cereals**				
Sourdough bread	Wheat, rye, corn, rice	*S. cerevisiae, S.bayanus, Lactobacillus* spp., *Lactococcus* spp., *Weissella* spp.	Organic acids and aromatic compounds production.	Comasio et al. (2020)
Red yeast rice	Rice	*Monascus purpureus*	Production of pigments and statins	Fukami et al. (2021)
**Dairy products**				
Cheese	Any kind of milk	*Lactococcus lactis*, *Lactobacillus* spp., *Streptococcus* spp. *Penicillium* sp.	Fatty acids oxidation.	Zheng et al. (2021)
Kefir	Cow, goat or ewe milk	*Saccharomyces kefir, Torula kefir, Lactobacillus caucasius*	Acid–alcoholic fermentation. Exopolysaccharides production.	Prado et al (2015)
Yogurt	Any kind of milk	*Streptococcus thermophiles, Lactobacillus delbrueckii* ssp. *bulgaricus*	Rapid fermentation.	Dan et al. (2019)
**Fruit, vegetables and soy-based**				
Kimchi and sauerkraut	Cabbage and other vegetables	*Leuconostoc mesenteroides, Lactobacillus sakei, Weisella koreensis*	Fermentation in high salinity. Production of bacteriocins.	Zabat et al. (2018); Dimidi et al. (2019)
Miso	Soybeans	*Aspergillus oryzae, Tetragenococcus halophilus, Zygosaccharomyces rouxii*	Fermentation in high salinity.	Allwood et al. (2021)
Tempeh	Soybeans	*Rhizopus oligosporus, R. oryzae*	Production of vitamins (B2, B6, nicotinic acid, nicotinamide).	Ahnan-Winarno et al. (2021)
Vinegar	Fruits or grains	*S. cerevisiae; Zygosaccharomyces spp., Acetobacter* spp.	Alcoholic and acetous fermentation.	Li et al. (2015)
**Meat-based**				
Ham, sausages	Different types of meat	*Lactobacillus spp., Pediococcus spp., Debaryomyces hansenii*	High production of bacteriocins, proteases and lipases.	Kumar et al. (2017)
Surströmming	Herring	*Alkalibacterium* spp.*, Carnobacterium* spp., *Tetragenococcus halophilus*	Production of volatile trimethylamine and sulphur compounds.	Belleggia et al. (2020)

* source considers the original source of the fermentation culture or spontaneous fermentation, not an inoculum.

### Domestication and tool-box creation for non-model organisms

Databases with whole genome data are growing constantly (Kodama et al., 2012). The reason for this, besides the dropping sequencing costs, is metagenomics studies. Metagenomics is the approach to sequence complex samples, rather than single organisms, to reconstruct communities of whole ecosystems (Ghurye et al., 2016; Lui et al., 2021; Zhang et al., 2021). However, besides the growing sequence data, the number of well-established model organisms in laboratories is limited. Research would benefit by broadening our molecular understanding of various, different organisms. Microbes from fermentation cultures are of particular interest based on their abilities to produce, enhance and preserve food. Isolation of individual microbes of a community can be achieved with standard cultivation techniques in media mimicking the fermentation process. A respective example workflow for microbe isolation and its domestication is given in Figure 1B. If one aims to isolate microbes it is important to be familiar with the Nagoya protocol which is an attempt to limit biopiracy (Smith et al., 2017). Once microbes of interest are isolated, the first step is their classification based on sequencing genetic markers (i.e., 16S RNA, internal transcribed spacer (ITS)) followed by whole genome sequencing if required. The combination of short- and long-read sequencing allows sequencing and assembly of any microbial genome (Bashir et al., 2012; Moss et al., 2020). One reason for this is that computational tools have become accessible and user-friendly (Ejigu and Jung, 2020; Jung et al., 2020). Sequencing genomes and performing *de novo* genome assembly is now affordable and achievable within a matter of days. Genome sequencing data can be supported with transcriptome data to enable accurate genome annotation (Prasad et al., 2017). However, a transcriptome sample always represents gene expression in one particular condition, not representing all transcripts, which may be the current major bottleneck in understanding genomes. In the future, complete genome assembly and annotation from metagenomic samples will further advance, resulting in more data in sequence databases (Yang et al., 2021). This sequence space will be of great value to understand enzymes and enzyme complexes and investigate their potential application, especially in the context of larger numbers of complete genomes available (De Filippis et al., 2020). While biological and biochemical characterization remains a bottleneck, laboratory automation in the form of biofoundries helps to accelerate research in this regard (Hillson et al., 2019; Farzaneh and Freemont, 2021).

Genetic engineering of communities is challenging due to multiple factors, but more and more organisms are becoming genetically accessible (Johns et al., 2016; McCarty and Ledesma-Amaro, 2019; Tsoi et al., 2019). To engineer non-model microbes, one must understand the organism to a greater extent and there are still challenges in the engineering of non-model organisms besides the recent advances in genetic engineering tools (Yan and Fong, 2017; Ren et al., 2020). However, comparing the identified microbe with already known, related species, may give hints as to which types of techniques may be successful for genetic engineering approaches. Being able to perform efficient genetic engineering is important to make a microorganism viable for future synthetic biology applications. The initial step is the transfer and stable incorporation of DNA sequences into the microbial genome in an ideally marker-free procedure. Once an efficient transformation procedure is established researchers can make use of the power of synthetic biology. One of the advantages of synthetic biology is the use of modular tool-boxes allowing quick and reliable construction of reusable DNA part libraries based on well-characterized genetic parts (Kelwick et al., 2014). A technology which has been widely adopted to achieve this is Golden Gate cloning, which relies on the use of type IIS restriction enzymes and allows modular cloning (Engler et al., 2008; Werner et al., 2012). Individual parts can be combined into transcriptional units to build complex pathways and it seems a size limit has not yet been reached. Researchers are able to construct whole synthetic chromosomes in mega-base ranges (Schindler et al., 2018; Zumkeller et al., 2018). In combination with laboratory automation, new organisms can be domesticated and explored. Different constructs can be built and screened, and deletion or interference libraries can be constructed to engineer an organism to understand its biology and biochemistry or for its application in industry.

In recent years studies were proposed and made towards direct and indirect improvement of agriculture, food quality, and sustainability, ideally creating a circular bioeconomy, but many challenges remain (Marvik and Philp, 2020). Genetic engineering has become highly precise allowing almost any change in genomes and synthetic biology allows researchers to make opportunities out of these challenges (El Karoui et al., 2019). Today, the organism of choice to tackle a challenge no longer needs to be haploid; CRISPR-based engineering allows efficient engineering of diploid and polyploid organisms (Knott and Doudna, 2018; Lian et al., 2018). Many yeast strains underwent complex genomic variations during their industrial domestication, resulting in complex, polyploid strains. This is common for example in brewing yeasts (Gallone et al., 2016). However, such genome alterations can cause sterility and limit breeding programs. Engineering such strains with CRISPR-based tools has an advantage as microbial diploid and polyploid phenotypes might be lost if forced to become haploid, should they be viable at all. Meanwhile, a recent publication highlights a novel non-GMO technique to unlock the functional potential of polyploid yeasts allowing its application in the food industry under the current legislation (Mozzachiodi et al., 2022).

### Future food generated by engineered microbes

Domesticating a microbe and generating an easy-to-use synthetic biology tool-box can facilitate the engineering of individual organisms and potentially communities, and opens new perspectives for future foods. In Table 2, we summarize a list of current products and compounds which production is based in the use of microbes engineered with synthetic biology tools. The main advantages for all these products include less consumption of water, less land as they would be produced by fermentation in bioreactors, no animal use, and some of them include enhancements in the organoleptic and nutritional properties.

**TABLE 2:** Products available or expected to enter the market in the near future produced by engineered microorganisms.

Product	Application	Reference
Bacterial cellulose	Food additive, nutritional supplement, packaging	Singh et al. (2020); Zhong (2020)
Benzoic acid	Food preservative	Luo and Lee. (2020)
Carotenoids: Astaxanthin	Nutritional supplement, food pigment	Basiony et al. (2022)
Carotenoids: Lycopene	Nutritional supplement, food pigments	Shi et al. (2019); Wang et al. (2020); Wang et al. (2022)
Casein	Protein for animal-free engineered milk	Patent: US20170273328A1
Exopolysaccharides	Food additives for enhancing texture and preservation	Boels et al. (2003); Prete et al. (2021) Patent: WO2015011266A1
Fatty acids	Production of oils and food additives	Marella et al. (2018); Liu et al. (2021); Sharma and Yazdani. (2021)
Leghemoglobin	Appearance, flavor and aroma enhancement of meat analogs	Acuna and Poblete-Castro. (2022) Patent: US20170349906A1
Muconic acid	Precursor for bioplastics production	Choi et al. (2020)
Polyhydroxyalkanoates (PHAs)	Packaging - Bioplastic	Zheng et al. (2020); Chaudhary et al. (2022)
Steviol glucosides	Next generation stevia sweeteners	Olsson et al. (2016); Xu et al. (2022)
Sweet-tasting proteins	Non-caloric sweeteners	Joseph et al. (2019); Bilal et al. (2022)

Beyond engineering microbes, much research has also been done to improve food quality itself by the production of supplements and compounds. Such is the case of Golden Rice, genetically engineered to produce β-carotene with the goal of overcoming vitamin A deficiencies, which might be one of the widely known yet controversial developments of genetically modified foods as a strategy for fighting malnutrition (Ye et al., 2000; Enserink, 2008; Stokstad, 2019). Importantly, Golden Rice does not solve the cause of malnutrition, society must step in to guarantee a balanced and sustainable diet for everyone. Further, placing genetically engineered plants into the field has always been restricted and highly controversial, especially in Europe, although the UK recently announced intentions to change its policy (Ledford, 2021). However, genetically engineered crops, like all other crops, have the drawback of requiring sufficient agricultural land, fertilizers and pesticides. In addition, genetically engineered crops pose a potential threat for biodiversity. Researchers may tackle these risks and challenges by replacing crops with microbes. The reason for this is that the desired product can be produced in fermentation vessels, which can be installed in almost any location. Fermentation vessels have a reasonable footprint, are not seasonal, do not require sunlight and therefore do not compete with nature and housing space. Further, it has been shown that various microbes like *Escherichia coli* and *S*. *cerevisiae* can be modified to produce various carotenoids in high yields (Shi et al., 2019; Wang et al., 2020; Basiony et al., 2022). Exemplary key products for future foods which currently are, or in the near future will be, produced based on synthetic biology principles are highlighted in table 2.

An example where researchers replaced plants with microbes is in the engineering of industrial brewing yeast to produce some of the flavor molecules needed in hopped beers (Denby et al., 2018). Hops are water-intensive crops with underlying seasonal quality changes. Engineered yeast strains for brewing purposes can be simply distributed and potentially produce consistent flavor profiles. Another example is the microbial production of milk and meat replacements (Table 2). Agriculture is one of the driving factors of climate change, with the dairy and cattle industries particularly producing large quantities of methane emissions. Researchers are on the path to produce alternative milk products through fermentation of plant-based material, and synthetic biology may offer additional alternatives in the future (Tangyu et al., 2019). The same holds true for the replacement of meat (Rubio et al., 2020). Already today, mycoproteins from non-genetically engineered *Fusarium venenatum,* formed into various meat alternatives are sold under the trademark Quorn (Trinci, 1992). The isolated fungus originated from soil samples in the 1960s where alternative food sources were investigated to tackle “*the world’s flagging supply of protein foods*”. This organism has since been domesticated in industrial scale fermentations. In recent years researchers started to explore strain optimization, applying CRISPR/Cas9 genome editing rather than random mutagenesis (Wilson and Harrison, 2021). Filamentous fungi in general are promising candidates to solve future food challenges (Strong et al., 2022). In the future genetic engineering will be able to not only improve products and make them more sustainable, but also to build ideal hosts suitable for future food production based on microbes for example from traditional fermentations. There are many advantages of using microbes as food or future food producers: they do not need large spaces and they usually do not need light. Microbes have the potential to allow on-demand, decentralized food production with consistent nutritional properties. 

Water is becoming a limiting resource on Earth, but there are microbes from many traditional fermentation cultures which are salt tolerant and might be ideal chassis for the production of future foods based on sea water. Synthetic biology can do much more than enhancing individual products. Synthetic biology aims to tackle problems caused by climate change and manmade pollution by seeing CO_2_ and plastic waste as alternative feedstocks (Bar-Even, 2016; Schwander et al., 2016; Sadler and Wallace, 2021).

### Synthetic biology for alternative feedstocks and biomaterials

Future food has challenges besides producing food itself. There is a need for alternatives for preserving, sustainable packaging and the resources needed for biomass production. In order to propagate and grow microbes at an industrial scale, suitable feedstocks are mandatory. Classical feedstocks compete with food-chain supplies, and therefore researchers are investigating alternative feedstocks. Alternative feedstocks should be cheap, easy to produce and should not cause competition with the classical food-chain. Resources from waste streams, for example spent brewers grain, pomace or organic waste, may be suitable feedstocks but have high variations between batches and seasons. Alternative roads would be the implementation of synthetic pathways allowing assimilation of alternative carbon sources. In recent years researchers have looked into the possibility of C_1_ compound assimilation, and in particular the assimilation of CO_2_, methanol and formate have sparked interest (Yishai et al., 2016; Claassens et al., 2018; Cotton et al., 2020; Fabarius et al., 2021; Jiang et al., 2021; Marcellin et al., 2022). Genetic engineering generally allows the modification of microbial hosts, enabling them to assimilate alternative, sustainable carbon sources. These projects now need to prove that they are scalable. This would be a major step towards a sustainable bioeconomy. Other alternative feedstocks like plastic waste are being explored and researchers are making progress in terms of identification, characterization and engineering of PET degrading enzymes (PETase) (Tournier et al., 2020; Sonnendecker et al., 2022). *Idionella sakaiensis* is a microbe where the complete degradation and assimilation of PET was discovered for the first time (Yoshida et al., 2016). Recent research shows post-consumer plastic treated with PETase under specific conditions can convert PET into terephthalate (TA) which can for example be converted by genetically engineered *Escherichia coli* cells to produce vanillin (Sadler and Wallace, 2021). There might be a day where genetically engineered microbes can build substantial biomass on a reasonable timescale from PET degradation. It was shown that TA can be used as feedstock for bacterial production of polyhydroxyalkanoates (PHAs) which can serve as bioplastic and are a sustainable alternative to PET based on their superior degradation properties (Kenny et al., 2008). Those abilities could be transferred to other microbes with synthetic biology tools including microbes from traditional fermentation processes and use PET as feedstock.

Future food challenges extend beyond production of food itself, with transportation and packaging also representing major challenges. Currently, one of the major sources of plastic waste is the packaging material of food. Moving away from single-use packaging towards reusable packaging would be already a big step. However, this is not always feasible however, this is not always feasible, but thankfully biological degradable plastics are emerging alternatives (Chen and Patel, 2012). This makes sustainable biomaterials a very important factor for future food challenges. Synthetic biology is offering innovative solutions to tackle these challenges of future food as well. Researchers are working on various biomaterials ranging from bioplastics, to spider silk, to cellulose-based materials and many more (Le Feuvre and Scrutton, 2018). Bioplastics already have an application in real life while other materials are still under development. The potential of materials to produce, for example, alternatives to petroleum-based packaging material is enormous. Especially if consumers move away from single-use packaging, the potential of biological materials is a considerable opportunity. Cellulose, which is naturally produced by microbes, is a promising biomaterial and has many applications (Zhong, 2020). Bacterial cellulose might one day be used to produce sustainable packaging for the food industry (Nesic et al., 2019). Inventors are exploring the potential of synthetic wood generated from the cellulose material of the kombucha industry in another example of the upcycling of waste streams with origin from fermentation cultures.

Researchers are able to perform sophisticated genetic engineering in increasing numbers of microbes. While most of the time a rather limited set of model organisms is applied, nature has much more to offer. Most likely there are organisms in nature which will perform much better in given tasks compared to any engineered *E. coli* or *S. cerevisiae* strain. Microbes from traditional fermentation procedures are a treasure trove in which to identify novel organisms which may perform desired tasks with only little effort in genetic engineering. There are opportunities for the production, enhancement and preservation of food, but also for biomaterials and alternative feedstocks to build a circular bioeconomy, with synthetic biology offering a suitable tool-box with which to achieve these goals.

## Conclusion and perspectives

For many reasons future food will not be produced as it is done today. The current methods of agriculture are not sustainable and are harmful to the Earth. In moving towards future food, many challenges will be addressed - some of which are already known and others which will become apparent during the transition. However, these challenges represent opportunities and synthetic biology in combination with traditional techniques might be one approach to tackle them. One of the major challenges will be the opinion of the consumer, who would have to be convinced that, at least to a certain proportion, food will be produced by engineered microorganisms. Microbes from traditional food fermentation procedures may provide the ideal chassis for synthetic biology by balancing tradition and modern technologies. This may sound futuristic but there are already many examples of products from engineered microbes used for medical purposes such as vaccines or drug precursors. So why not apply engineered microbes to create a circular bioeconomy?

An obvious challenge, but at the same time a research accelerator for future food, lies in the exploration of space. Humankind seems set to again become an explorer, and aims to install settlements on the Moon and Mars. The efforts of space exploration will greatly benefit from synthetic biology and its application. With the establishment of a settlement far away from Earth, food supplies will rely on the progress of synthetic biology to create future foods. Will these developments be beneficial for everyone, similar to the development of Teflon now essential part of non-stick pans common in many kitchens?

Why is the acceptance of products from genetically engineered microbes or crops to feed humanity low? To the knowledge of the authors, no human being has died so far because of the consumption of food originating from genetically engineered sources, but according to the United Nations every day 25,000 people, including more than 10,000 children, die from starvation and malnutrition! From the authors’ perspective, the major challenge might not be sustainable food production: it is convincing policy makers, politicians and consumers to make sustainable choices and be open towards novel technologies such as precision gene editing to optimize production and enhance products. The authors acknowledge that vigorous testing of such engineered organisms and their products is compulsory and there must be regulations, however, one of the advantages of microorganisms is that they are kept under controlled environments and may even contain engineered safety switches to ensure they do not interfere with biodiversity if exposed to the environment. Nature has been suffering from decades of traditional agriculture and industrialization. Engineered microorganisms should be explored further to tackle future food challenges and ultimately establish a circular bioeconomy.

## Data Availability

The original contributions presented in the study are included in the article/Supplementary Material, further inquiries can be directed to the corresponding author.
